# Neo-BCV: A Novel Bacterial Complex Vaccine Reshaping the Gut Microbiota to Enhance the Antitumor Immune Response

**DOI:** 10.3390/vaccines14040310

**Published:** 2026-03-30

**Authors:** Hairu Yang, Huiqin Zhu, Zilong Zhu, Lingyun Man, Qingfeng Pang, Tao Lu, Kecheng Xu, Zhenyi Wang, Peihua Lu

**Affiliations:** 1 Department of Oncology, Wuxi People’s Hospital Affiliated to Nanjing Medical University, Wuxi Medical Center, Nanjing Medical University, Wuxi 214023, China; 19930556829@163.com (H.Y.); winny073@163.com (H.Z.); zilongzhu_njmu@126.com (Z.Z.); 2 School of Medicine, Jiangnan University, Wuxi 214122, China; zhizhiweizhizhi777@163.com (L.M.); qfpang@jiangnan.edu.cn (Q.P.); 3 School of Life Science, Beijing University of Chinese Medicine, Beijing 100029, China; taolu@bucm.edu.cn; 4 Oncology Department, National Key Clinical Specialty (Oncology), Biomedical Translational Research Institute, Fuda Cancer Hospital, Jinan University, Guangzhou 510632, China; xukc@vip.163.com; 5 National Research Centre for Translational Medicine at Shanghai, State Key Laboratory of Medical Genomics, Research Unit of Hematologic Malignancies Genomics and Translational Research of Chinese Academy of Medical Sciences, Shanghai Institute of Hematology, Ruijin Hospital, Shanghai Jiao Tong University School of Medicine, Shanghai 200025, China; 13661734438@163.com

**Keywords:** tumor vaccines, non-small cell lung cancer, *Lactobacillus reuteri*, taurocholic acid, tumor immunity

## Abstract

Background: Lung cancer is the most common malignancy worldwide and has the highest mortality rate. Although therapeutic approaches have improved over recent years, the clinical efficacy of lung cancer treatment remains limited. Therefore, there is an urgent need to develop novel and effective immunotherapeutic strategies for lung cancer. Methods: In this study, we constructed a novel bacterial complex vaccine (Neo-BCV, hereafter referred to as BCV) and investigated its anti-tumor effects and underlying mechanisms in a murine lung cancer model. We further explored the role of the gut microbiota, bile acid metabolism, and T-cell function in BCV-mediated anti-tumor immunity. In addition, we performed a preliminary evaluation of the clinical safety of BCV in human subjects. Results: BCV treatment significantly enhanced the infiltration of CD4^+^ and CD8^+^ T cells into the tumor immune microenvironment and promoted the secretion of anti-tumor effector molecules. Mechanistically, BCV markedly increased the abundance of *Lactobacillus reuteri* (*L. reuteri*) in the gut microbiota and reduced serum levels of taurocholic acid (TCA). Further experiments confirmed that *L. reuteri* directly degrades TCA, and decreased TCA levels restored the effector functions of CD4^+^ and CD8^+^ T cells. Conclusions: This study demonstrates that BCV remodels the gut microbiota and enhances anti-tumor immunity by regulating the *L. reuteri*–TCA axis to restore T-cell function. This mechanism provides a new strategy for improving the tumor immune microenvironment and supports further investigation and development of BCV as a therapeutic candidate for lung cancer.

## 1. Introduction

Since the start of the 21st century, global cancer incidence and mortality rates have been on a gradual rise. Among all malignancies, lung cancer accounts for the highest proportions of both new cases and deaths, imposing a substantial social burden and causing significant economic losses [1,2]. Conventional treatment strategies for lung cancer include surgical resection, chemotherapy, and radiotherapy; however, these approaches are often associated with poor prognosis and a high recurrence rate [1]. Over the past decade, remarkable advances have been made in cancer immunotherapy [3]. Among the various immunotherapeutic modalities, immune checkpoint inhibitors (ICIs) stand out as the most representative [4]. Nevertheless, the clinical efficacy of immune checkpoint blockade therapy in lung cancer remains relatively limited, with an overall response rate of less than 20% among patients [5]. Therefore, there is an urgent need to explore novel therapeutic strategies.

The utilization of bacteria for tumor therapy boasts a long-standing history [6]. In 1891, William Coley administered a mixture of heat-inactivated *Streptococcus pyogenes* and *Serratia marcescens* to patients with inoperable osteosarcoma, and observed cases of significant tumor volume reduction or even complete remission [7]. Termed “Coley’s toxin”, this bacterial mixture was used to treat over one thousand cancer patients in the subsequent three decades, with some patients carrying advanced sarcoma achieving long-term survival [8]. Retrospective data from 128 patients treated with Coley’s toxin indicated that their survival rates were comparable to those achieved with modern conventional therapies [9]. Another controlled trial further re-evaluated the toxin and demonstrated its ability to mediate antitumor effects [10]. Moreover, a phase I clinical trial demonstrated that Coley’s toxin could induce robust production of immunomodulatory cytokines, which may play a role in tumor regression [11]. However, during treatment, Coley’s toxin was also observed to cause fatal complications such as embolism, acute nephritis, hemorrhage, and overdose, highlighting its unstable safety profile, which ultimately prevented its widespread clinical adoption [12,13].

In recent years, with the advancement of synthetic biology, engineered bacteria modified via synthetic biology approaches have exhibited reduced toxicity and acquired tumor-targeting capabilities [14]. Based on this, we have optimized the formulation of Coley’s toxin and developed a novel bacterial complex vaccine (Neo-BCV, hereafter referred to as BCV), using safer and more potent components. This vaccine is a composite of multiple bacteria and toxins, including *Bordetella pertussis*, typhoid/paratyphoid bacilli, *Staphylococcus aureus*, diphtheria toxin, and tetanus toxin. A series of studies have demonstrated that these six bacterial strains and their respective toxins can effectively activate multiple Toll-like receptors (TLRs), thereby triggering both innate and adaptive immune responses [15,16,17,18,19]. Furthermore, compared with Coley’s toxin, these components exert fewer adverse effects on the human body. By combining these ingredients, we hypothesize that they can more efficiently activate anti-tumor immunity while significantly enhancing safety profiles.

Our preliminary experiments have shown that BCV can effectively activate the host immune system, promote the trafficking and infiltration of anti-tumor immune cells into tumor lesions, and confer long-lasting immune effects [20]. In the present study, we will further investigate the mechanism of action of BCV to clarify the specific role of this bacterial mixture in activating anti-tumor immunity.

The gut microbiota has been demonstrated to be closely associated with tumor immunity [21]. Among the metabolites of the gut microbiota, bile acids have garnered attention owing to their interactions with immune cells. A growing body of evidence indicates that microbially derived bile acids can modulate the host immune response [22,23].

In our study, we observed a significant upregulation of *Lactobacillus reuteri* (*L. reuteri*) in the gut of mice treated with BCV. Concurrently, the levels of effector molecules secreted by T cells were markedly increased. Further investigations revealed that *L. reuteri* restored the effector functions of CD4^+^ and CD8^+^ T cells by reducing the level of taurocholic acid (TCA).

## 2. Methods

### 2.1. Details of Bacteria, Cells, Animals, and Study Participants

*Lactobacillus reuteri* was provided by the Guangdong Microbial Culture Center (Guangzhou, China).

The Lewis lung adenocarcinoma (LLC) cell line was purchased from Wuhan Procell Life Science & Technology Co., Ltd. (Wuhan, China).

Male C57BL/6 mice (4–6 weeks old) were obtained from Nanjing GemPharmatech Co., Ltd. (Nanjing, China). All animal experiments were performed in the Lung Transplantation Animal Laboratory of Wuxi People’s Hospital. The experimental protocol was approved by the Laboratory Animal Management Committee of Nanjing Medical University (Approval Code: DL2022034; Approval Date: 18 October 2022).

Peripheral blood samples were obtained from patients with lung cancer in Wuxi Kaiyi Hospital, Medical Treatment Combination of Wuxi People’s Hospital. This study was approved by the Clinical Research Ethics Committee of Wuxi People’s Hospital (KY23009) and was registered with ClinicalTrials.gov (Trial registration number: NCT05747339). The individuals were informed about this study and gave consent before specimen collection.

### 2.2. Preparation of BCV, FMT and L. reuteri Bacterial Cultures

The bacterial composite vaccine (BCV) comprises 6 heat-inactivated bacterial components: (1) *Bordetella pertussis* (9 billion CFU/mL), (2) *Corynebacterium diphtheriae* toxin (20 Lf/mL), (3) *Clostridium tetani* toxin (5 Lf/mL), (4) *Salmonella Typhi* (300 million CFU/mL), (5) *Salmonella Paratyphi* A/B (150 million CFU/mL each), and (6) 10% *Staphylococcus aureus* solution (1 billion CFU/mL). All bacterial strains were obtained from the National Institutes for Food and Drug Control. Bacterial culture and medium preparation services were commissioned to Zhejiang Weixin Biological Pharmaceutical Co., Ltd. (Hangzhou, China). Additionally, glucan (0.2 g/mL) was incorporated as an adjuvant.

Preparation of FMT: FMT samples were prepared by collecting fecal pellets from mice in the negative control (NC) and BCV treatment groups. The fecal samples were mixed with sterile normal saline at a concentration of 50 mg/mL, vortexed thoroughly, and centrifuged to collect the supernatant for subsequent use.

Preparation of *L. reuteri* suspension: *L. reuteri* was inoculated into De Man, Rogosa, and Sharpe (MRS) liquid medium and cultured anaerobically at 37 °C until the optical density (OD_600_) reached 1.6. The bacterial cells were then collected by centrifugation, washed, and resuspended in phosphate-buffered saline (PBS). The concentration was adjusted to 1 × 10^9^ CFU/mL for subsequent use.

### 2.3. Animal Models and Treatment Options

To establish a subcutaneous tumor model, LLC cells (1 × 10^6^) were injected subcutaneously into the right axillary region, approximately 1 cm below the armpit, of C57BL/6 mice. Each treatment began when the tumor became palpable, usually 4 days after inoculation. Notably, before the fecal transplantation experiment, mice need to be administered antibiotics by oral gavage daily from day 0 to day 4 (vancomycin 50 mg/mL, imipenem/cilastatin 25 mg/mL, neomycin 10 mg/mL, Amphotericin 1 mg/mL).

For BCV treatment, mice were divided into two groups: the NC group and the BCV group. Four days after tumor cell inoculation, 200 μL of PBS or BCV was injected subcutaneously into the dorsal region of the mice twice a week for two weeks.

For fecal microbiota transplantation or *L. reuteri* transplantation, mice were divided into three groups: Trans-NC, Trans-BCV, and Trans-*L. reuteri* groups. For the Trans-NC and Trans-BCV groups, a total of 100 μL of fecal suspension was administered to the mice by oral gavage. For the Trans-*L. reuteri* group, 200 μL of *Lactobacillus reuteri* suspension (2 × 10^8^ CFU) was administered by oral gavage. All groups received gavage twice a week.

For the TCA administration groups, mice were divided into three groups: NC, BCV, and BCV + TCA. Tumor-bearing mice were orally gavaged with TCA at a dose of 100 mg/kg once daily for two weeks.

For the combined BCV and αPD-1 treatment, mice were divided into four groups: NC, BCV, αPD-1, and BCV + αPD-1. Mice received BCV twice a week for two weeks. Following BCV administration, αPD-1 (20 mg/kg) was administered by intraperitoneal injection twice a week.

### 2.4. In Vivo Evaluation of Antitumor Efficacy

To evaluate antitumor efficacy, tumor volume was measured every five days. After 18 days of treatment, blood was collected via orbital bleeding, feces were collected, and tumors and spleens were excised for subsequent experiments. The spleens and tumors were photographed and weighed. Then, mice were anesthetized with tribromoethanol (0.2 mL/20 g body weight) and euthanized by cervical dislocation.

To explore the immune mechanism, tumors were collected. Harvested subcutaneous tumors were minced into small pieces using sterile scissors. After passing through a 70-μm cell strainer, the resulting cell suspension was resuspended in RPMI-1640 medium and carefully overlaid onto a 40% Percoll solution. An 80% Percoll solution was then gently layered beneath the 40% Percoll solution to form a density gradient. Following centrifugation, lymphocytes at the interphase were carefully collected and washed with cold PBS buffer. Subsequently, cells were harvested and stained for surface antigens with anti-mouse CD45, anti-mouse CD3, anti-mouse CD4, anti-mouse CD8, anti-mouse CD19, anti-mouse CD44, and anti-mouse CD62L monoclonal antibodies (mAbs) for 30 min at 4 °C in the dark. After fixation and permeabilization, intracellular staining was performed with anti-mouse IFN-γ, anti-mouse TNF-α, anti-mouse granzyme B, and anti-mouse perforin mAbs for 30 min at 4 °C in the dark. Cells were then washed twice with permeabilization buffer and analyzed by flow cytometry using a Navios flow cytometer (Beckman Coulter). Data analysis was performed with FlowJo Version 10.5.0 software.

Additionally, immunohistochemistry was performed on mouse tumor tissues. Paraffin-embedded mice tumor tissues were cut into 5-μm sections to undergo IHC staining. The sections were stained with antibodies anti-mouse CD4 (1:1000, Abcam, Boston, MA, USA), CD8 (1:500, Bioss, Beijing, China), CD19 (1:500, Diagbio, Hangzhou, China), visualized via HRP-conjugated polyclonal goat anti-mouse or anti-rabbit IgG (Solarbio). Positive cells were detected by confocal microscopy (Carl Zeiss LSM880 with NLO & Airyscan).

### 2.5. Quantitative Detection of Bile Acids

To further verify that BCV down-regulates the level of taurocholic acid in mouse serum, we collected the serum of treated mice. Fresh supernatant of bacterial culture medium was snap frozen in liquid nitrogen and then kept at −80 °C. Levels of total, conjugated, unconjugated and specific bile acids (BAs) were measured at the Shanghai Metabolome Institute-Wuhan (Wuhan, China) using an ultra-high-performance liquid chromatography/electrospray ionization tandem mass spectrometry (UPLC-ESI-MS/MS) system (1290-6470, Agilent Technologies, Inc., Santa Clara, CA, USA). In brief, samples (1 μL) were separated using a Kinetex Core–Shell 2.6 μm C18 column (100 × 2.1 mm, 2.6 μm, Phenomenex Inc. Torrance, CA, USA) equipped with Kinetex 2.6 μm Minibore Security Guard Ultra Cartridges (Phenomenex Inc., Torrance, CA, USA) at 45 °C. The mobile phases consisted of A (water with 0.005% HCOOH, *v*/*v*) and B (acetonitrile with 0.005% HCOOH, *v*/*v*). The stepwise elution gradient process was as follows: 1.23% B to 33% B for 2 min; 2.33% B to 34% B for 4 min; 3.34% B to 70% B for 5 min. The flow rate was 0.6 mL/min. Mass spectrometry (MS) detection of BAs was conducted in negative ion mode. Fragmentor and product ions for every BA were optimized through the direct infusion of available BA standards to improve detection sensitivity. Due to the low proportion of some bile acids in samples, we chose [2]- as the product ion to promote sensitivity under multiple reaction monitoring (MRM) scan mode. Data acquisition and analysis were performed with Mass Hunter software (Agilent Technologies, Inc., Santa Clara, CA, USA) [24].

### 2.6. TCA Deconjugation Using L. reuteri

To verify the ability of *Lactobacillus reuteri* (*L. reuteri*) to degrade TCA, we performed an in vitro co-culture of TCA and *L. reuteri*. The abundance of *L. reuteri* was diluted to 10^7^ CFU/mL in a freshly prepared DeMan, Rogosa and Sharpe (MRS) medium. TCA was then added to each culture to obtain a final concentration of 4 μM. The cultures were incubated in an anaerobic chamber at 37 °C for 48 h. The culture supernatant was collected by centrifugation and assayed for changes in TCA and CA levels.

### 2.7. Analysis of the Immune Effects of TCA on Human PBMC In Vitro

Peripheral blood mononuclear cells (PBMCs) from lung cancer patients were isolated by Ficoll-Hypaque density gradient centrifugation. Freshly isolated human PBMCs were cultured in RPMI 1640 complete medium supplemented with 10% fetal bovine serum (FBS), 100 U/mL penicillin, and 100 μg/mL streptomycin. PBMCs were seeded into 96-well plates and incubated with various concentrations of taurocholic acid (TCA) (50/100 μM) for 24 h. Subsequently, the cells were stimulated with phorbol 12-myristate 13-acetate (PMA), Ionomycin, brefeldin A (BFA), and monensin for 5 h. Intracellular staining of CD4+ or CD8+ T cells for IFN-γ, TNF-α, granzyme B, and perforin was assessed by flow cytometry. The specific procedures were consistent with those for animal tumor flow cytometry.

### 2.8. Tumor Tissue RNA Extraction and Sequencing

Total RNA was isolated from tumor specimens with TRIzol^®^ Reagent (Invitrogen) (Carlsbad, CA, USA), following the manufacturer’s standard protocols precisely. To eliminate genomic DNA contamination, the RNA preparations were further treated with DNase I (TaKaRa).

The integrity and quality of RNA were evaluated using an Agilent 2100 Bioanalyzer (Agilent Technologies) (Santa Clara, CA, USA) while RNA concentration was determined by a NanoDrop ND-2000 spectrophotometer (NanoDrop Technologies) (Wilmington, DE, USA). Only high-quality RNA samples that satisfied the required criteria were employed for the construction of sequencing libraries: OD_260_/OD_280_ ratio of 1.8–2.2, OD_260_/OD_230_ ratio ≥2.0, RNA Integrity Number (RIN) ≥6.5, 28S/18S rRNA ratio ≥1.0, and total RNA amount >10 μg.

Using 1 μg of total RNA for each sample, transcriptome libraries for RNA sequencing were constructed with the Illumina TruSeq™ RNA Sample Preparation Kit (Illumina, San Diego, CA, USA).

After quantification with a TBS380 fluorometer, the paired-end libraries were subjected to high-throughput sequencing (150 bp × 2 read length) at Shanghai BIOZERON Co., Ltd. (Shanghai, China). The raw sequencing reads have been deposited in the NCBI Sequence Read Archive (SRA) database under the accession number SRP525811.

### 2.9. Fecal Sample and Tumor Tissue DNA Extraction, PCR Amplification and Sequencing

Fecal samples and tumor tissues were obtained for DNA extraction. Microbial DNA was isolated from mouse fecal specimens and tumor tissues with the E.Z.N.A.^®^ Stool DNA Kit and Tissue DNA Kit (Omega Bio-tek, Norcross, GA, USA), following the manufacturer’s standard protocols. The V4-V5 hypervariable region of the bacterial 16S ribosomal RNA gene was amplified by PCR. Amplification was carried out using primers 341F 5′-CCTAYGGGRBGCASCAG-3′ and 806R 5′-GGACTACNNGGGTATCTAAT-3′, in which an 8-base unique barcode was assigned to each individual sample. PCR was conducted in triplicate in a 20 μL reaction mixture consisting of 4 μL 5 × FastPfu Buffer, 2 μL of 2.5 mM dNTPs, 0.8 μL of each primer (5 μM), 0.4 μL FastPfu Polymerase, and 10 ng template DNA. PCR amplicons were separated on 2% agarose gels and recovered using the AxyPrep DNA Gel Extraction Kit (Axygen Biosciences, Union City, CA, USA) in accordance with the manufacturer’s instructions. The purified amplicons were quantified with a Qubit^®^ 3.0 fluorometer (Life Invitrogen) (Carlsbad, CA, USA), and twenty-four distinct barcoded samples were pooled at an equimolar ratio. The pooled DNA mixture was used to prepare an Illumina paired-end library following the manufacturer’s standard genomic library construction pipeline. Subsequently, the amplicon library was subjected to paired-end sequencing (2 × 250) on a high-throughput sequencing platform (Shanghai BIOZERON Biotech. Co., Ltd.) (Shanghai, China) according to standard procedures. Fecal samples and tumor tissues were obtained for DNA extraction. The mice had not received any antibiotic treatment before sample collection. The following steps were conducted by BIOZERON Biotechnology Co., Ltd. (Shanghai, China). Raw sequencing reads were deposited in the NCBI Sequence Read Archive (SRA) under accession number SRP525811 [25,26].

OTUs were clustered with a 97% similarity cutoff using Usearch (version 10) and chimeric sequences were identified and removed using UCHIME. The phylogenetic affiliation of each 16S rRNA gene sequence was analyzed by uclust algorithm (version 1.2.22q) against the silva (Release 138.1) 16S rRNA database (http://www.arb-silva.de) using a confidence threshold of 80%. Following the generation of operational taxonomic units (OTUs), 16S rDNA sequencing data were analyzed on the CFViSA platform, which integrates a professional microbiome analysis pipeline and nearly 80 analytical tools covering basic sequence processing, data visualization, and statistical analysis for amplicon sequencing data [26].

### 2.10. Untargeted Metabolomic Screen

Fresh mouse serum was snap frozen in liquid nitrogen and then kept at −80 °C. The following steps were conducted by BIOZERON Biotechnology Co., Ltd. (Shanghai, China). The samples (100 μL) were placed in the EP tubes and resuspended with prechilled 80% methanol and 0.1% formic acid by well vortex. The samples were incubated on ice for 5 min and centrifuged at 15,000× *g* at 4 °C for 20 min. An aliquot of the supernatant was diluted with LC-MS grade water to a final concentration of 53% methanol. The mixture was then transferred to a new Eppendorf tube and centrifuged again at 15,000× *g* at 4 °C for 20 min. The resulting supernatant was subsequently injected into the LC-MS/MS system for analysis. UHPLC-MS/MS analyses were carried out using a Vanquish UHPLC system (ThermoFisher, Braunschweig, Germany) coupled with an Orbitrap Q Exactive™ HF-X mass spectrometer (ThermoFisher, Germany) at BIOZERON Biotechnology Co., Ltd. (Shanghai, China). Sample separation was performed on a Hypesil Gold column (100 × 2.1 mm, 1.9 μm) with a 17-min linear gradient at a flow rate of 0.2 mL/min. For the positive polarity mode, eluent A was 0.1% formic acid (FA) in water and eluent B was methanol. For the negative polarity mode, eluent A was 5 mM ammonium acetate (pH 9.0) and eluent B was methanol. The solvent gradient program was set as follows: 2% B for 1.5 min; 2–100% B over 12.0 min; 100% B for 14.0 min; 100–2% B within 14.1 min; and 2% B until 17 min. The Q Exactive™ HF-X mass spectrometer was operated in both positive and negative ionization modes with a spray voltage of 3.2 kV, capillary temperature of 320 °C, sheath gas flow rate of 40 arb, and auxiliary gas flow rate of 10 arb. Raw data acquired from UHPLC-MS/MS were processed using Compound Discoverer 3.1 (CD3.1, ThermoFisher) for peak alignment, peak selection, and metabolite quantification. The data supporting the findings of this study have been deposited in the CNGB Sequence Archive (CNSA) of the China National GeneBank Database (CNGBdb) under accession number CNP0006099 [27].

### 2.11. Statistical Analysis

Statistical analyses were performed using GraphPad Prism version 8.0 (GraphPad Software). One-way ANOVA, paired *t*-test, and unpaired *t*-test were used to evaluate statistical differences between groups. *p* < 0.05 was regarded as statistically significant.

## 3. Results

### 3.1. BCV Ameliorates the Tumor Immune Microenvironment and Suppresses Tumor Growth in Mice

**Figure 1:**
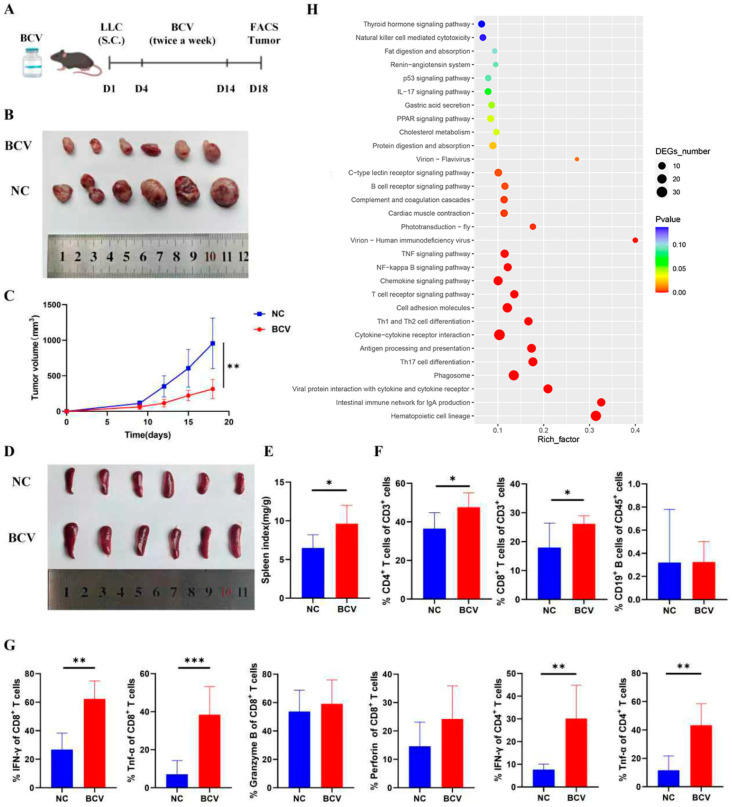
BCV Suppresses Tumor Growth and Ameliorates the Tumor Immune Microenvironment in Mice. (**A**) Schematic of the treatment protocol. Lewis lung cancer (LLC) cells (1 × 10^6^ cells/200 μL) were subcutaneously (s.c.) injected into C57BL/6 mice. Four days later, mice were administered 200 μL of PBS or BCV twice weekly for 2 weeks. Mice receiving PBS were designated as the negative control (NC) group, while those receiving BCV were designated as the BCV group. (**B**) Representative macrographs of tumors from mice with or without BCV treatment. (**C**) Tumor growth curves of mice treated with or without BCV (*n* = 6 per group). (**D**) Representative macrographs of spleens from mice with or without BCV treatment. (**E**) Analysis of the spleen weight-to-body weight ratio (*n* = 6 per group). (**F**) Quantification of CD3^+^CD4^+^ T cells, CD3^+^CD8^+^ T cells, and CD3^−^CD19^+^ B cells in tumors from BCV-treated (*n* = 6) and untreated (*n* = 6) mice. (**G**) Quantification of the expression of the indicated molecules in CD4^+^ and CD8^+^ T cells. (**H**) KEGG pathway enrichment analysis of differentially enriched genes in tumors from BCV-treated (*n* = 3) and untreated (*n* = 3) mice. Unpaired *t*-test. * *p* < 0.05, ** *p* < 0.01, *** *p* < 0.001.

To evaluate the in vivo antitumor efficacy of BCV, C57BL/6 mice were subcutaneously injected with either PBS (control) or BCV twice weekly, starting after subcutaneous implantation of Lewis lung cancer (LLC) cells (Figure 1A). The administration route and dose of BCV were determined following verification of its immunostimulatory activity and vaccination safety.

Compared with the control group (NC), mice treated with BCV exhibited significantly smaller tumor volumes (Figure 1B,C). BCV was well tolerated in mice, with no weight loss or hepatic toxicity observed (Supplementary Figure S1A,B). Mice receiving BCV injections showed a marked increase in spleen size and spleen index (Figure 1D,E), indicating that BCV effectively activates the peripheral immune system in mice.

Next, we investigated the tumor immune microenvironment (TIME) in these mice. Immunohistochemical staining revealed the infiltration of CD4^+^ T cells, CD8^+^ T cells, and CD19^+^ B cells at the tumor site in both NC and BCV groups (Supplementary Figure S1C). To further characterize TIME changes, we performed fluorescence-activated cell sorting (FACS) analysis (Supplementary Figure S1D). Compared with the NC group, the proportion of CD4^+^ and CD8^+^ T cells in the TIME of BCV-treated mice was significantly elevated, whereas the proportion of B cells remained unchanged (Figure 1F). Additionally, in-depth analysis of cellular effector functions demonstrated that both CD4^+^ and CD8^+^ T cells in the tumors of BCV-treated mice secreted significantly higher levels of IFN-γ and TNF-α, whereas the secretion of Granzyme B and Perforin by CD8^+^ T cells showed no significant difference (Figure 1G).

We subsequently performed RNA sequencing (RNA-seq) analysis on tumors isolated from the two groups of mice, and the results revealed distinct gene expression signatures (Supplementary Figure S1E). Kyoto Encyclopedia of Genes and Genomes (KEGG) pathway enrichment analysis demonstrated that BCV activated multiple immunity-related signaling pathways, including the TNF signaling pathway, NF-κB signaling pathway, chemokine signaling pathway, T cell receptor signaling pathway, B cell receptor signaling pathway, and antigen processing and presentation pathway (Figure 1H).

BCV Induces a Beneficial Shift in Gut Microbiota Composition, Thereby Retarding Tumor Growth.

We next investigated the underlying cause of tumor immune microenvironment (TIME) changes in BCV-treated mice. Fecal samples were collected from mice to extract bacterial DNA, and taxonomic profiling was conducted via 16S ribosomal RNA (rRNA) gene sequencing.

At the operational taxonomic unit (OTU) level, no significant differences were observed between the two groups in terms of observed species, Chao1 index, or Simpson index (Supplementary Figure S2A). However, the Shannon index was significantly higher in the BCV group than in the NC group, indicating greater alpha diversity of the gut microbiota in BCV-treated mice compared to NC mice. Additionally, a clustered heatmap illustrating Bray–Curtis dissimilarity among individual samples at the OTU level revealed no distinct gut microbiota variation profiles between mice of different groups (Supplementary Figure S2B,C).

We further investigated whether the gut microbiota composition of BCV-treated mice differed from that of NC mice. First, we evaluated the overall gut microbiota landscape of individual mice in both groups. Results revealed significant differences in bacterial community composition between the two groups across distinct taxonomic levels (Supplementary Figure S3A–F).

**Figure 2:**
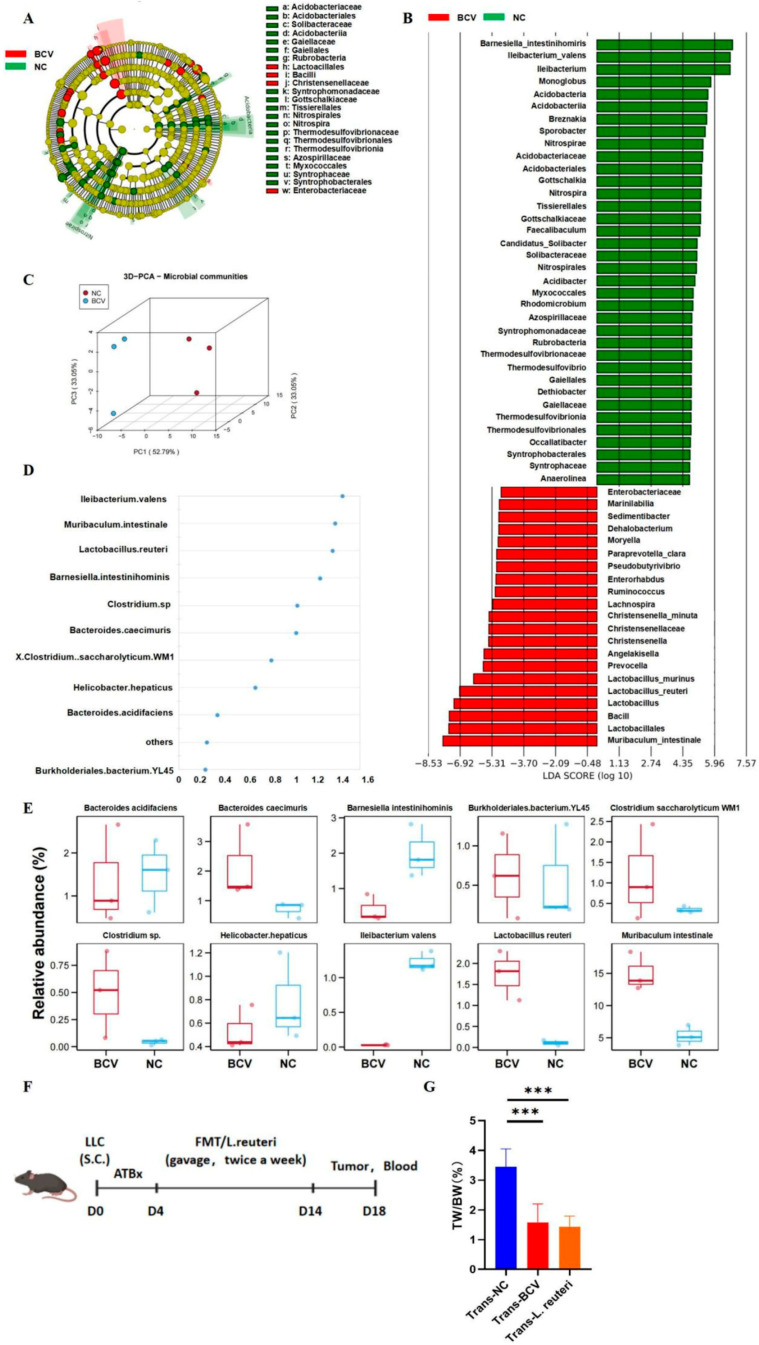
Alterations in Gut Microbiota Composition Between NC and BCV-Treated Mice. (**A**) Taxonomic cladogram generated by linear discriminant analysis effect size (LEfSe) illustrating taxonomic associations in microbial communities from NC and BCV-treated mice. Each node represents a specific taxonomic level. Yellow nodes indicate taxonomic features with no significant difference between the two groups; green nodes represent taxa more abundant in NC mice; red nodes represent taxa more abundant in BCV-treated mice. (**B**) Linear discriminant analysis (LDA) scores of differentially abundant microbial features between NC and BCV-treated mice. Features were selected based on the criteria of LDA score > 2 and *p* < 0.05. (**C**) Three-dimensional principal component analysis (3D-PCA) score plot showing the species abundance distribution of fecal samples from NC mice (red dots) and BCV-treated mice (blue dots). (**D**) Variable importance in projection (VIP) scores ranking the discriminative capacity of different taxa between the NC and BCV groups. (**E**) Inter-group comparisons of the relative abundance of the top 10 bacterial taxa. (**F**) Schematic diagram of fecal microbiota transplantation or *Lactobacillus reuteri* (*L. reuteri*) transplantation. (**G**) Analysis of the tumor weight-to-body weight ratio (*n* = 5 per group). One-way analysis of variance (ANOVA). *** *p* < 0.001.

To validate these observations, we performed linear discriminant analysis effect size (LEfSe) [28]—a high-dimensional classification comparison method—to determine whether bacterial communities differed significantly between NC and BCV mice (Figure 2A,B). At the genus level, a notable increase in the abundance of *Lactobacillus* was observed in BCV-treated mice. Three-dimensional principal component analysis (3D-PCA) showed distinct clustering patterns of samples from NC and BCV mice (Figure 2C). Variable importance in projection (VIP) scoring for gut microbiota indicated that *Lactobacillus reuteri* (*L. reuteri*) made a significant contribution to the separation of the two groups (Figure 2D). Additionally, comparisons of the relative abundance of the top 10 bacterial taxa revealed that *L. reuteri* abundance was significantly higher in BCV-treated mice than in NC mice (Figure 2E). Collectively, these data demonstrate that BCV modulates the gut microbiota composition of mice, with a marked upregulation of *L. reuteri* in particular.

Accumulating evidence highlights a unique role of *L. reuteri* in antitumor therapy [29,30]. To further confirm whether the gut microbiota of BCV-treated mice exerts antitumor effects, we administered fecal samples from NC or BCV mice to LLC-bearing recipient mice via oral gavage (Figure 2F). Additionally, we directly transplanted *L. reuteri* into recipient mice to verify its specific antitumor activity; the three groups were designated as Trans-NC, Trans-BCV, and Trans-*L. reuteri*, respectively (Figure 2F). Notably, oral gavage with either *L. reuteri* or BCV-mouse feces resulted in retarded tumor growth (Figure 2G). Taken together, these findings confirm that the gut microbiota of BCV-treated mice—particularly *L. reuteri*—retards tumor progression in recipient mice.

### 3.2. BCV Downregulates Serum Taurocholic Acid Levels in Mice

Next, we investigated the specific mechanism by which gut microbiota exerts antitumor effects. Given the potential translocation of intestinal bacteria into tumors [31], we analyzed the composition of intratumoral bacteria in NC and BCV-treated mice via 16S rRNA gene sequencing. No significant differences were observed between the two groups in terms of observed species, Shannon index, Chao1 index, or Simpson index (Supplementary Figure S4A). Beta (β) diversity analysis also revealed no significant intergroup differences in intratumoral bacterial communities (Supplementary Figure S4B–D). Furthermore, intratumoral bacterial composition showed minimal changes across distinct taxonomic levels in both groups (Supplementary Figure S4E,F). Collectively, these results indicate that intratumoral bacterial communities did not differ substantially between the two groups.

**Figure 3:**
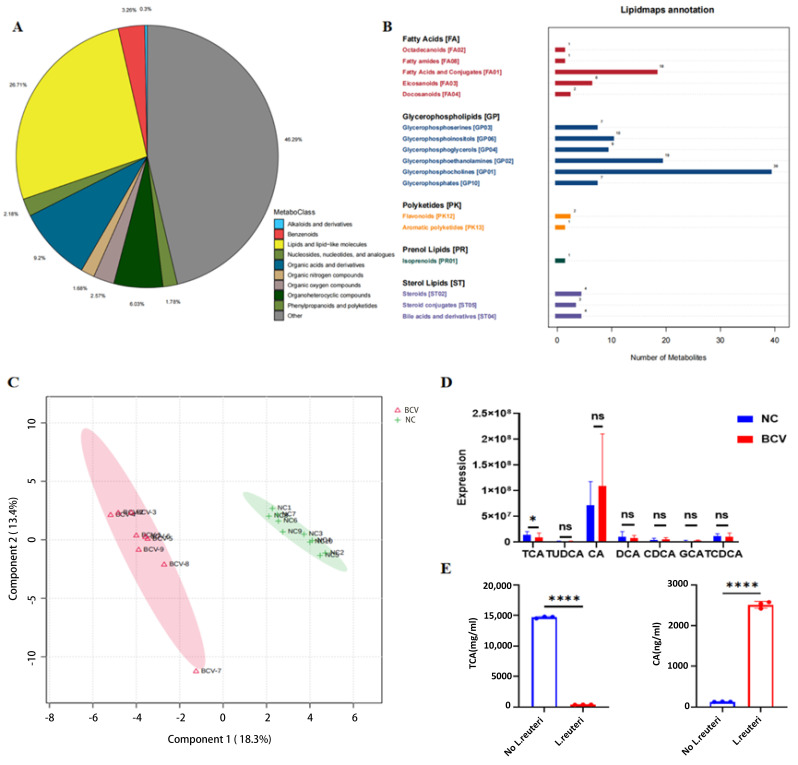
Reduced Serum Taurocholic Acid (TCA) Levels in BCV-Treated Mice. (**A**) Metabolomic analysis of serum metabolites from mice with or without BCV treatment. (**B**) LIPID MAPS annotation of serum lipids in negative ion mode. (**C**) Partial least squares discriminant analysis (PLS-DA) score plot showing the metabolite abundance distribution of serum samples from NC mice (*n* = 10, green) and BCV-treated mice (*n* = 9, red). (**D**) Relative abundance of bile acids (BAs) in NC and BCV-treated mice. (**E**) In vitro TCA deconjugation activity of *Lactobacillus reuteri* (*L. reuteri*). Abbreviations: CA, Cholic acid; CDCA, Chenodeoxycholic acid; DCA, Deoxycholic acid; GCA, Glycocholic acid; TCA, Taurocholic acid; TCDCA, Taurochenodeoxycholic acid; TUDCA, Tauroursodeoxycholic acid. Unpaired *t*-test. ns, not significant,* *p* < 0.05, **** *p* < 0.0001.

We then analyzed serum samples from NC and BCV-treated mice and performed an untargeted metabolomic screen using liquid chromatography–tandem mass spectrometry (LC-MS/MS) [32]. The identified metabolites were categorized and subjected to statistical analysis (Figure 3A). These metabolites were primarily classified as lipids and lipid-like molecules. We further conducted LIPID MAPS annotation, which identified fatty acids, glycerophospholipids, polyketides, prenol lipids, and sterol lipids in negative ion mode (Figure 3B). Notably, sterol lipids included several bile acids and their derivatives. Partial least squares discriminant analysis (PLS-DA) demonstrated significant differences in serum metabolite profiles between NC and BCV-treated mice (Figure 3C). We therefore focused on identifying key metabolites that modulate antitumor immunity.

In this study, the serum abundance of taurocholic acid (TCA) was significantly reduced in the BCV group (Figure 3D). Previous studies have reported that TCA impairs the effector functions of immune cells [33,34,35]. We hypothesized that the gut microbiota of BCV-treated mice enhances the functions of CD4^+^ and CD8^+^ T cells by reducing TCA levels, thereby mediating antitumor effects.

Bile salt hydrolases (BSHs) expressed by gut microbiota catalyze the cleavage of amide bonds in conjugated bile acids (CBAs), producing unconjugated bile acids (UBAs) [36]. A recent study has shown that *Lactobacillus* is a key bacterial genus encoding BSH [37]. We cultured *L. reuteri* in vitro to verify its ability to deconjugate TCA. After 48 h, targeted quantitative detection of TCA was conducted. Compared with the control group (without *L. reuteri*), the TCA level was significantly decreased in the *L. reuteri*-treated group—consistent with our hypothesis—while the level of cholic acid (CA; the corresponding hydrolysis product of TCA) was significantly increased (Figure 3E). These findings indicate that BCV effectively reduces serum TCA levels in mice by upregulating the abundance of *L. reuteri*.

Reducing Taurocholic Acid (TCA) Levels Restores the Effector Functions of CD4^+^ and CD8^+^ T Cells from Patients with Lung Cancer.

**Figure 4:**
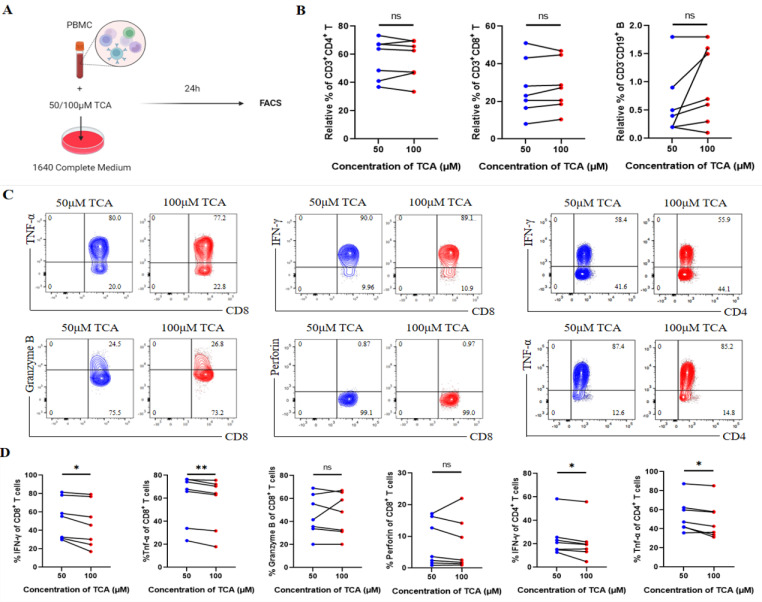
Low Taurocholic Acid (TCA) Concentrations Restore CD4^+^ and CD8^+^ T Cell Function in Patients with Lung Cancer. (**A**) Schematic diagram of the in vitro experimental design. (**B**) Proportions of CD3^+^CD4^+^ T cells, CD3^+^CD8^+^ T cells, and CD3^−^CD19^+^ B cells in peripheral blood mononuclear cells (PBMCs) from patients with lung cancer treated with low (50 μM, *n* = 7) or high (100 μM, *n* = 7) TCA concentrations. (**C**) Representative flow cytometry (FCM) plots showing the expression of the indicated molecules in CD3^+^CD4^+^ T cells and CD3^+^CD8^+^ T cells in each group. (**D**) Quantification of the expression levels of the indicated molecules in CD3^+^CD4^+^ T cells and CD3^+^CD8^+^ T cells. Paired *t*-test. ns, not significant, * *p* < 0.05, ** *p* < 0.01.

Subsequently, to investigate TCA’s regulatory role in the immune system, we performed in vitro co-culture experiments. Specifically, we evaluated the effects of different TCA concentrations on immune cells by stimulating freshly isolated peripheral blood mononuclear cells (PBMCs) from seven patients with lung cancer (Figure 4A). After 24 h of stimulation, cells were collected for fluorescence-activated cell sorting (FACS) analysis.

TCA concentrations did not alter the proportions of CD4^+^ T cells, CD8^+^ T cells, or B cells (Figure 4B). However, intracellular staining for IFN-γ, TNF-α, granzyme B, and perforin revealed that a low TCA concentration (50 μM) restored the cytokine production levels of CD4^+^ and CD8^+^ T cells compared to a high TCA concentration (100 μM) (Figure 4C,D). These results indicate that reducing TCA levels can partially restore the secretion of IFN-γ and TNF-α by CD4^+^ and CD8^+^ T cells from patients with lung cancer in vitro.

### 3.3. BCV Restores the Effector Functions of CD4^+^ and CD8^+^ T Cells In Vivo by Reducing Taurocholic Acid (TCA) Levels

**Figure 5:**
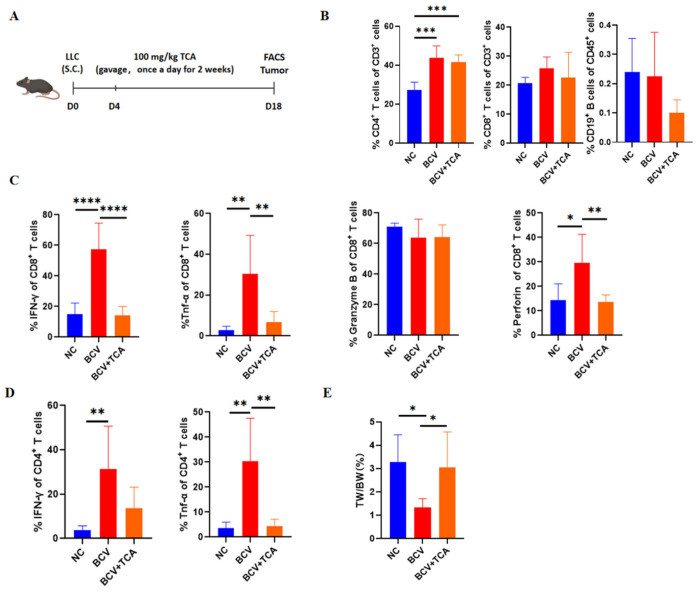
BCV Restores the Effector Functions of CD4^+^ and CD8^+^ T Cells In Vivo by Reducing Taurocholic Acid (TCA) Levels. (**A**) Schematic diagram of the in vivo experimental design. Once tumors became palpable, mice were administered TCA (100 mg/kg) via oral gavage once daily for 2 weeks. (**B**) Proportions of CD4^+^ T cells, CD8^+^ T cells, and B cells in tumors from NC (*n* = 5), BCV (*n* = 6), and BCV + TCA (*n* = 6) mice. (**C**,**D**) Quantification of the expression levels of the indicated molecules in CD4^+^ and CD8^+^ T cells. (**E**) Analysis of the tumor weight-to-body weight ratio. One-way analysis of variance (ANOVA). * *p* < 0.05, ** *p* < 0.01, *** *p* < 0.001, **** *p* < 0.0001.

To determine whether TCA reduction also restores the effector functions of CD4^+^ and CD8^+^ T cells in vivo, we administered TCA (100 mg/kg) daily to C57BL/6 mice for 2 weeks following subcutaneous tumor implantation (Figure 5A).

The FACS analysis of TIME shows the percentage of CD4^+^ and CD8^+^ T cells was significantly higher in the BCV group than in the NC group; however, TCA administration failed to counteract this increase (Figure 5B). Consistent with our previous findings (Figure 1D,E), CD4^+^ and CD8^+^ T cells in the BCV group produced higher levels of cytokines and cytotoxic molecules. Notably, TCA treatment antagonized these elevated cytokine levels, reducing them back to the low levels observed in the NC group (Figure 5C,D). Concurrently, the antitumor effects mediated by BCV were abrogated by TCA administration (Figure 5E).

Taken together, these results demonstrate that BCV enhances the effector functions of CD4^+^ and CD8^+^ T cells in mice by reducing serum TCA levels.

### 3.4. Combination Therapy with BCV and PD-1 Monoclonal Antibody Enhances Antitumor Efficacy in Mice

**Figure 6:**
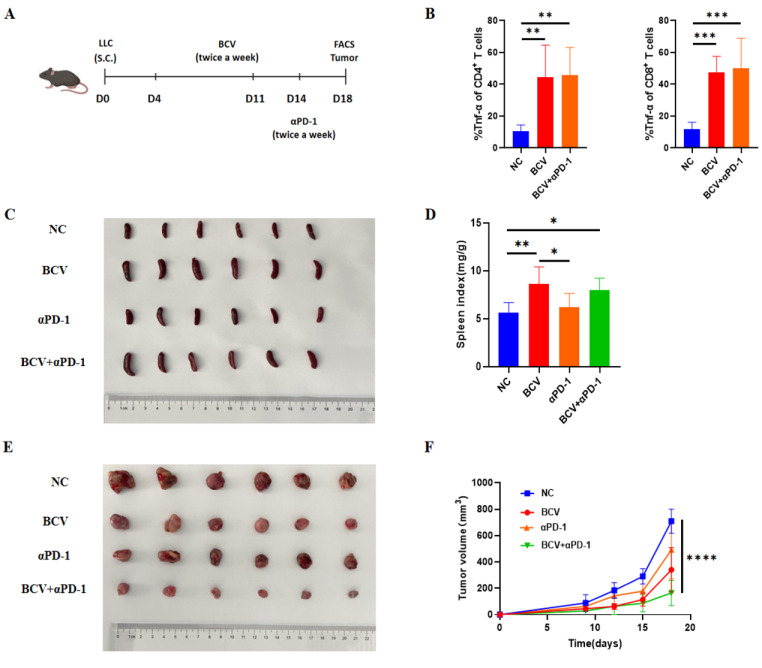
Combination of BCV and αPD-1 Enhances Immune Responses and Antitumor Efficacy in Mice. (**A**) Schematic diagram of the treatment protocol. Mice were administered αPD-1 (20 mg/kg) twice weekly, starting one week after BCV administration. (**B**) Quantification of TNF-α expression in CD4^+^ and CD8^+^ T cells (*n* = 6 per group). (**C**) Representative macrographs of spleens from mice in the control, BCV, αPD-1, and BCV + αPD-1 groups. (**D**) Analysis of the spleen weight-to-body weight ratio (*n* = 6 per group). (**E**) Representative macrographs of tumors from mice in the control, BCV, αPD-1, and BCV + αPD-1 groups. (**F**) Tumor growth curves of mice in the control, BCV, αPD-1, and BCV + αPD-1 groups (*n* = 6 per group). One-way analysis of variance (ANOVA). * *p* < 0.05, ** *p* < 0.01, *** *p* < 0.001, **** *p* < 0.0001.

In recent years, PD-1 monoclonal antibody therapy has been widely used for lung cancer treatment; however, its clinical efficacy remains relatively limited. To investigate whether BCV can enhance the efficacy of immunotherapy, we administered BCV to mice followed by treatment with an anti-PD-1 antibody (αPD-1) (Figure 6A).

In this study, significantly higher levels of TNF-α secreted by CD4^+^ and CD8^+^ T cells were detected in tumor tissues from BCV-treated and BCV + αPD-1-treated mice compared to NC mice (Figure 6B), which is consistent with our previous findings. Concurrently, BCV combined with αPD-1 therapy increased the spleen index and promoted peripheral immune activation (Figure 6C,D). Most importantly, the combination treatment further retarded tumor growth in mice (Figure 6E,F). These results demonstrate that combining BCV with immune checkpoint inhibitors (ICIs) exerts a potent synergistic effect on antitumor activity.

## 4. Discussion

The current treatment landscape for lung cancer remains fraught with significant challenges. Even with the widespread use of immune checkpoint inhibitors (ICIs), their efficacy remains highly limited—with a response rate of less than 20% in patients with advanced disease [38]. Additionally, the prohibitive cost of such treatments cannot be overlooked. Historically, Coley’s toxin attracted considerable attention for its notable efficacy in advanced cancer, yet its unclear mechanisms and safety concerns limited broader application [7]. Our study aimed to optimize the formulation of Coley’s Toxin and developed a novel bacterial complex vaccine, BCV, consisting of bacteria and bacterial toxins. As hypothesized, BCV effectively suppressed tumor growth in murine models. Furthermore, we elucidated the specific immune activation mechanism underlying BCV’s antitumor effects.

Numerous studies have established that the gut microbiota plays a pivotal role in regulating and shaping the host’s antitumor immune system [39,40], underscoring its status as a key modulator of antitumor immunity. Consistent with this framework, our study found that the abundance of *Lactobacillus reuteri* (*L. reuteri*) in the gut microbiota was significantly elevated in BCV-treated mice compared to control mice. Functional validation further confirmed that mice receiving fecal microbiota transplantation (FMT) from BCV-treated mice or direct *L. reuteri* transplantation exhibited marked antitumor effects. As a ubiquitous intestinal symbiont, *L. reuteri* is currently a focal point of microbiome research [41]. Notably, previous studies have demonstrated that *L. reuteri* can enhance the efficacy of immune checkpoint inhibitor (ICI) therapy by metabolizing tryptophan [29]. This prior finding motivated us to investigate the potential crosstalk between *L. reuteri* and host metabolomics—particularly the role of *L. reuteri*-derived metabolic alterations in mediating BCV’s antitumor effects.

Current studies have established that the gut microbiota and its derived metabolites mediate the functional regulation of immune cells [42]. Consistent with this, our results showed a significant reduction in serum taurocholic acid (TCA) levels in BCV-treated mice. A recent study has demonstrated that *Lactobacillus* species highly express bile salt hydrolases (BSHs)—enzymes that catalyze the degradation of conjugated bile acids (CBAs) [37]. Guided by this finding, we investigated the ability of *Lactobacillus reuteri* (*L. reuteri*) to degrade TCA in vitro. Our results revealed that *L. reuteri* significantly reduced TCA levels in the culture medium.

Emerging evidence indicates that gut microbiota-modified bile acids (BAs) can promote tumor growth by suppressing immune cell function [43]. We therefore hypothesized that BCV restores the effector functions of CD4^+^ and CD8^+^ T cells in vivo by downregulating TCA levels. This study confirmed this hypothesis: reducing TCA levels upregulated the secretion of effector molecules by CD4^+^ and CD8^+^ T cells, both in vitro (using peripheral blood mononuclear cells from lung cancer patients) and in vivo (murine models). Collectively, these findings delineate BCV’s antitumor mechanism: BCV upregulates *L. reuteri* abundance in vivo, which in turn reduces TCA levels, enhances the effector functions of CD4^+^ and CD8^+^ T cells, and ultimately exerts an antitumor effect—highlighting BCV’s unique value in tumor therapy.

Although our study demonstrated that BCV ameliorates the tumor immune microenvironment (TIME) and inhibits tumor growth, it has several limitations that warrant consideration. First, while we observed BCV-induced alterations in the murine gut microbiota, the long-term stability, even permanence, of these changes remains to be validated through dedicated longitudinal experiments. Second, as the study was conducted exclusively in murine models, the findings may not fully recapitulate BCV’s mode of action in humans. To address this translational gap, we are actively anticipating generating further insights using humanized models. Moreover, the specific molecular mechanism by which TCA inhibits T cell effector function remains unclear and requires further investigation. Meanwhile, preliminary clinical results indicate that BCV is well tolerated in patients and may activate dendritic cells and downstream immune responses. However, the number of patients was limited, and longer-term administration of BCV for extended monitoring of immune cell changes was not performed. These unresolved mechanisms of action will be the focus of our subsequent studies.

In conclusion, our study demonstrates that BCV modulates the intestinal microenvironment by upregulating the abundance of *Lactobacillus reuteri* (*L. reuteri*). In turn, *L. reuteri* enhances the effector functions of CD4^+^ and CD8^+^ T cells by downregulating taurocholic acid (TCA) levels, ultimately exerting an antitumor effect. This work not only reaffirms the therapeutic potential of bacterial-based therapies for tumors but also provides a feasible framework for elucidating the underlying mechanisms of such treatments, with preliminary clinical data supporting the consistency and safety.

## 5. Conclusions

This study successfully developed a novel bacterial composite vaccine (BCV) and systematically elucidated its mechanism of exerting anti-tumor effects by regulating the gut microbiota–metabolic axis. The research results showed that BCV can significantly upregulate the abundance of *Lactobacillus reuteri* in mice’s intestines. This bacterium degrades taurocholic acid (TCA) through its expressed bile salt hydrolase, thereby relieving TCA-mediated inhibition of effector functions of CD4^+^ and CD8^+^ T cells, ultimately reshaping the tumor immune microenvironment and inhibiting lung cancer growth.

The main innovations and contributions of this study are as follows: First, we modernized Coley’s toxin, which has been historically well-regarded but limited due to unclear mechanisms, developing a BCV formulation with defined components and clear mechanisms, providing a new direction for the development of bacterial-based tumor immunotherapy. Second, we first revealed the molecular mechanism by which BCV affects anti-tumor immunity by regulating specific gut commensal bacteria (*Lactobacillus reuteri*) and their metabolites (TCA), establishing a novel regulatory axis of ‘bacterial vaccine-gut microbiota–bile acid metabolism-T cell function’. Third, through bidirectional validation in peripheral blood mononuclear cells from lung cancer patients and mouse models, we confirmed the inhibitory effect of TCA on T cell effector functions and its potential as a therapeutic target.

In summary, this study not only provides a promising new strategy for lung cancer immunotherapy but also establishes a research paradigm for understanding the mechanisms of bacterial-based tumor immunotherapy, confirming the great potential of regulating the gut microbiota–metabolic–immune axis in tumor treatment.

Although our study demonstrates that BCV reshapes the gut microbiota and bile acid metabolism in mice, thereby improving the tumor immune microenvironment and suppressing tumor growth, it still has significant limitations. First, different tumor models, such as spontaneous tumor formation and tumor metastasis, need to be established to determine the generalizability of BCV across multiple tumor types. Second, since this study is limited to mice, the findings may not fully reflect the mode of action of BCV in humans. In addition, besides metabolites from the gut microbiota, BCV may also be involved in the regulation of anti-tumor immunity through other pathways. In fact, we are simultaneously investigating the immunological training effects of BCV on antigen-presenting cells and macrophages. Finally, the specific mechanism by which tricarboxylic acid inhibits T cell effector function remains to be further studied. These additional olfactory mechanisms will be further explored in sub-quantum studies.

## Data Availability

Data and materials are available from the corresponding author on reasonable request (10.6084/m9.figshare.30590132). Further inquiries can be directed to the corresponding author.

## Supplementary Materials

The following supporting information can be downloaded at: https://www.mdpi.com/article/10.3390/vaccines14040310/s1, Figure S1: BCV has no side effects in mice; Figure S2: Profiling of the gut microbiota in NC and BCV mice;; Figure S3: The taxonomic composition of the gut microbiota in NC and BCV mice model; Figure S4:There is little difference in the intratumoral bacteria between NC and BCV mice; Table S1: Key resources tablee.

## References

[1] Lahiri A., Maji A., Potdar P.D., Singh N., Parikh P., Bisht B., Mukherjee A., Paul M.K. (2023). Lung cancer immunotherapy: Progress, pitfalls, and promises. Mol. Cancer.

[2] Leiter A., Veluswamy R.R., Wisnivesky J.P. (2023). The global burden of lung cancer: Current status and future trends. Nat. Rev. Clin. Oncol..

[3] Denault M.-H., Melosky B. (2021). Immunotherapy in the First-Line Setting in Wild-Type NSCLC. Curr. Oncol..

[4] Yu H., Boyle T.A., Zhou C., Rimm D.L., Hirsch F.R. (2016). PD-L1 Expression in Lung Cancer. J. Thorac. Oncol..

[5] Wang M., Herbst R.S., Boshoff C. (2021). Toward personalized treatment approaches for non-small-cell lung cancer. Nat. Med..

[6] Sepich-Poore G.D., Zitvogel L., Straussman R., Hasty J., Wargo J.A., Knight R. (2021). The microbiome and human cancer. Science.

[7] McCarthy E.F. (2006). The toxins of William B. Coley and the treatment of bone and soft-tissue sarcomas. Iowa Orthop. J..

[8] Coley W.B. (1893). The treatment of malignant tumors by repeated inoculations of erysipelas. With a report of ten original cases. Am. J. Med. Sci..

[9] Richardson M.A., Ramirez T., Russell N.C., Moye L.A. (1999). Coley toxins immunotherapy: A retrospective review. Altern. Ther. Health Med..

[10] Johnston B.J., Novales E.T. (1962). Clinical effect of Coley’s toxin. II. A seven-year study. Cancer Chemother. Rep..

[11] Karbach J., Neumann A., Brand K., Wahle C., Siegel E., Maeurer M., Ritter E., Tsuji T., Gnjatic S., Old L.J. (2012). Phase I clinical trial of mixed bacterial vaccine (Coley’s toxins) in patients with NY-ESO-1 expressing cancers: Immunological effects and clinical activity. Clin. Cancer Res..

[12] Pelner L., Fowler G.A. (1959). Host-tumor antagonism. XIV. Sarcoma of the soft tissues treated by bacterial toxins: Unsuccessful series. J. Am. Geriatr. Soc..

[13] Coley W.B. (1910). The Treatment of Inoperable Sarcoma by Bacterial Toxins (the Mixed Toxins of the Streptococcus erysipelas and the Bacillus prodigiosus). Proc. R. Soc. Med..

[14] Gurbatri C.R., Arpaia N., Danino T. (2022). Engineering bacteria as interactive cancer therapies. Science.

[15] Pietrocola G., Arciola C.R., Rindi S., Di Poto A., Missineo A., Montanaro L., Speziale P. (2011). Toll-like receptors (TLRs) in innate immune defense against *Staphylococcus aureus*. Int. J. Artif. Organs.

[16] Carlin G., Viitanen E. (2005). In vitro pyrogenicity of the diphtheria, tetanus and acellular pertussis components of a trivalent vaccine. Vaccine.

[17] Kurtz J.R., Goggins J.A., McLachlan J.B. (2017). Salmonella infection: Interplay between the bacteria and host immune system. Immunol. Lett..

[18] Gillard J., Suffiotti M., Brazda P., Venkatasubramanian P.B., Versteegen P., de Jonge M.I., Kelly D., Bibi S., Pinto M.V., Simonetti E. (2024). Antiviral responses induced by Tdap-IPV vaccination are associated with persistent humoral immunity to *Bordetella pertussis*. Nat. Commun..

[19] Arpaia N., Godec J., Lau L., Sivick K.E., McLaughlin L.M., Jones M.B., Dracheva T., Peterson S.N., Monack D.M., Barton G.M. (2011). TLR signaling is required for Salmonella typhimurium virulence. Cell.

[20] Zhu Z., Chu Z., Fei F., Wu C., Fei Z., Sun Y., Chen Y., Lu P. (2025). Neo-BCV: A Novel Bacterial Liquid Complex Vaccine for Enhancing Dendritic Cell-Mediated Immune Responses Against Lung Cancer. Vaccines.

[21] Dai Z., Zhang J., Wu Q., Fang H., Shi C., Li Z., Lin C., Tang D., Wang D. (2020). Intestinal microbiota: A new force in cancer immunotherapy. Cell Commun. Signal..

[22] Collins S.L., Stine J.G., Bisanz J.E., Okafor C.D., Patterson A.D. (2023). Bile acids and the gut microbiota: Metabolic interactions and impacts on disease. Nat. Rev. Microbiol..

[23] Matson V., Chervin C.S., Gajewski T.F. (2021). Cancer and the Microbiome-Influence of the Commensal Microbiota on Cancer, Immune Responses, and Immunotherapy. Gastroenterology.

[24] Lin H., An Y., Tang H., Wang Y. (2019). Alterations of Bile Acids and Gut Microbiota in Obesity Induced by High Fat Diet in Rat Model. J. Agric. Food Chem..

[25] Bao K., Wang M., Liu L., Zhang D., Jin C., Zhang J., Shi L. (2023). Jinhong decoction protects sepsis-associated acute lung injury by reducing intestinal bacterial translocation and improving gut microbial homeostasis. Front. Pharmacol..

[26] Zhou S., Zhu Z., Chen X., Pu Q., Liu S., Luo Y., Shao Y., Sun Y., Sun X., Shang C. (2025). Study on the effects of urea addition on the fermentation quality, nitrogen metabolism, microbial community, and metabolic characteristics of cotton strawlage. Front. Microbiol..

[27] He Q., Guo K., Wang L., Xie F., Zhao Q., Jiang X., He Z., Wang P., Li S., Huang Y. (2023). Tannins amount determines whether tannase-containing bacteria are probiotic or pathogenic in IBD. Life Sci. Alliance.

[28] Ferreira R.M., Pereira-Marques J., Pinto-Ribeiro I., Costa J.L., Carneiro F., Machado J.C., Figueiredo C. (2018). Gastric microbial community profiling reveals a dysbiotic cancer-associated microbiota. Gut.

[29] Bender M.J., McPherson A.C., Phelps C.M., Pandey S.P., Laughlin C.R., Shapira J.H., Sanchez L.M., Rana M., Richie T.G., Mims T.S. (2023). Dietary tryptophan metabolite released by intratumoral *Lactobacillus reuteri* facilitates immune checkpoint inhibitor treatment. Cell.

[30] Hu C., Xu B., Wang X., Wan W., Lu J., Kong D., Jin Y., You W., Sun H., Mu X. (2023). Gut microbiota-derived short-chain fatty acids regulate group 3 innate lymphoid cells in HCC. Hepatology.

[31] Fu A., Yao B., Dong T., Chen Y., Yao J., Liu Y., Li H., Bai H., Liu X., Zhang Y. (2022). Tumor-resident intracellular microbiota promotes metastatic colonization in breast cancer. Cell.

[32] Tintelnot J., Xu Y., Lesker T.R., Schönlein M., Konczalla L., Giannou A.D., Pelczar P., Kylies D., Puelles V.G., Bielecka A.A. (2023). Microbiota-derived 3-IAA influences chemotherapy efficacy in pancreatic cancer. Nature.

[33] Cai J., Sun L., Gonzalez F.J. (2022). Gut microbiota-derived bile acids in intestinal immunity, inflammation, and tumorigenesis. Cell Host Microbe.

[34] Ma C., Han M., Heinrich B., Fu Q., Zhang Q., Sandhu M., Agdashian D., Terabe M., Berzofsky J.A., Fako V. (2018). Gut microbiome-mediated bile acid metabolism regulates liver cancer via NKT cells. Science.

[35] Xun Z., Lin J., Yu Q., Liu C., Huang J., Shang H., Guo J., Ye Y., Wu W., Zeng Y. (2021). Taurocholic acid inhibits the response to interferon-α therapy in patients with HBeAg-positive chronic hepatitis B by impairing CD8+ T and NK cell function. Cell. Mol. Immunol..

[36] Foley M.H., Walker M.E., Stewart A.K., O’fLaherty S., Gentry E.C., Patel S., Beaty V.V., Allen G., Pan M., Simpson J.B. (2023). Bile salt hydrolases shape the bile acid landscape and restrict Clostridioides difficile growth in the murine gut. Nat. Microbiol..

[37] Song Z., Cai Y., Lao X., Wang X., Lin X., Cui Y., Kalavagunta P.K., Liao J., Jin L., Shang J. (2019). Taxonomic profiling and populational patterns of bacterial bile salt hydrolase (BSH) genes based on worldwide human gut microbiome. Microbiome.

[38] Mazieres J., Drilon A., Lusque A.B., Mhanna L., Cortot A., Mezquita L., Thai A.A., Mascaux C., Couraud S., Veillon R. (2019). Immune checkpoint inhibitors for patients with advanced lung cancer and oncogenic driver alterations: Results from the IMMUNOTARGET registry. Ann. Oncol..

[39] Wastyk H.C., Fragiadakis G.K., Perelman D., Dahan D., Merrill B.D., Yu F.B., Topf M., Gonzalez C.G., Van Treuren W., Han S. (2021). Gut-microbiota-targeted diets modulate human immune status. Cell.

[40] Choi S.-C., Brown J., Gong M., Ge Y., Zadeh M., Li W., Croker B.P., Michailidis G., Garrett T.J., Mohamadzadeh M. (2020). Gut microbiota dysbiosis and altered tryptophan catabolism contribute to autoimmunity in lupus-susceptible mice. Sci. Transl. Med..

[41] Han J.-X., Tao Z.-H., Wang J.-L., Zhang L., Yu C.-Y., Kang Z.-R., Xie Y., Li J., Lu S., Cui Y. (2023). Microbiota-derived tryptophan catabolites mediate the chemopreventive effects of statins on colorectal cancer. Nat. Microbiol..

[42] Cervantes-Barragan L., Chai J.N., Tianero M.D., Di Luccia B., Ahern P.P., Merriman J., Cortez V.S., Caparon M.G., Donia M.S., Gilfillan S. (2017). *Lactobacillus reuteri* induces gut intraepithelial CD4+CD8αα+ T cells. Science.

[43] Cong J., Liu P., Han Z., Ying W., Li C., Yang Y., Wang S., Yang J., Cao F., Shen J. (2024). Bile acids modified by the intestinal microbiota promote colorectal cancer growth by suppressing CD8+ T cell effector functions. Immunity.

