# Supplementary material for: Neo-BCV: A Novel Bacterial Complex Vaccine Reshaping the Gut Microbiota to Enhance the Antitumor Immune Response
Source: Vaccines (Basel). 2026 Mar 30;14(4):310. doi: 10.3390/vaccines14040310 (PMC13120426; doi:10.3390/vaccines14040310)
Supplement: Supplementary file 1 [file vaccines-14-00310-s001.zip › vaccines-4173864-supplementary.pdf]

# Supplementary Materials for

## Neo-BCV: A Novel Bacterial Complex Vaccine Reshaping the Gut Microbiota to Enhance the Antitumor Immune Response

This file includes:

Supplementary Figure S1. BCV has no side effects in mice.

Supplementary Figure S2. Profiling of the gut microbiota in NC and BCV mice.

Supplementary Figure S3. The taxonomic composition of the gut microbiota in NC and BCV mice model.

Supplementary Figure S4. There is little difference in the intratumoral bacteria between NC and BCV mice.

Supplementary Table S1. Key resources table.

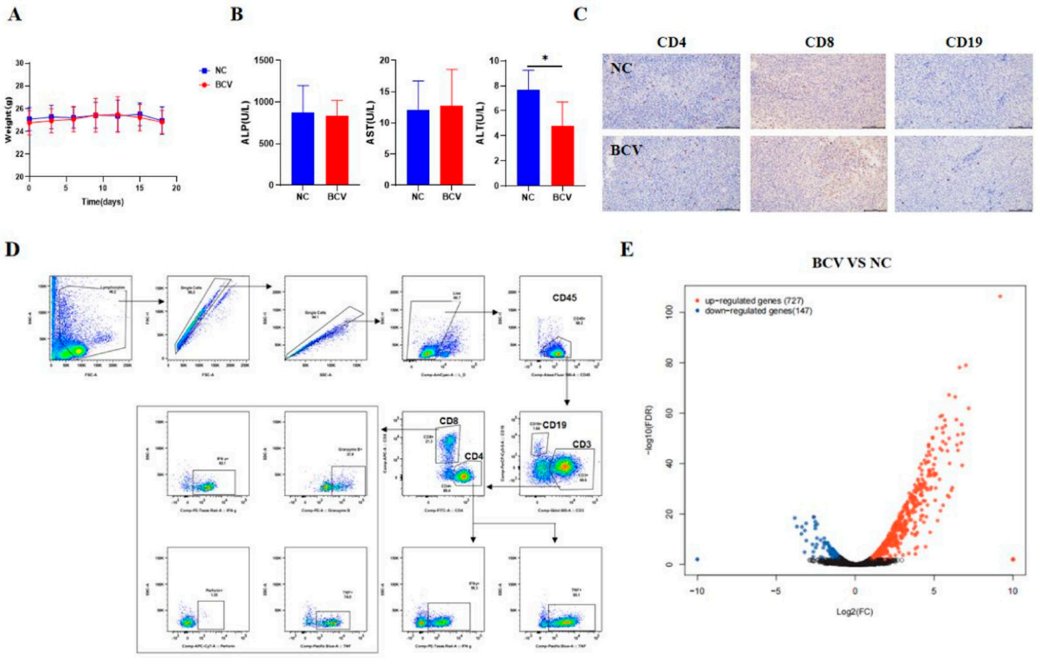


**Supplementary Figure S1. BCV has no side effects in mice. (A)** Body weight curves of mice treated with or without BCV($n$=6). **(B)** Hepatic function indexes in various mice($n$=6). **(C)** Representative plots and statistical analysis of CD4, CD8 and CD19 immunohistochemical staining of tumor sections in various mice, as indicated ($n$=4). **(D)** Schematic plot of flow gating strategy. **(E)** Volcano plot showing

differential gene expression in tumors from NC ($n$=3) mice compared to BCV ($n$=3) mice by RNA-seq. Colored dots (blue and red) represent genes with a log2FC>1 and FDR<0.05. Unpaired t test. *p < 0.05.

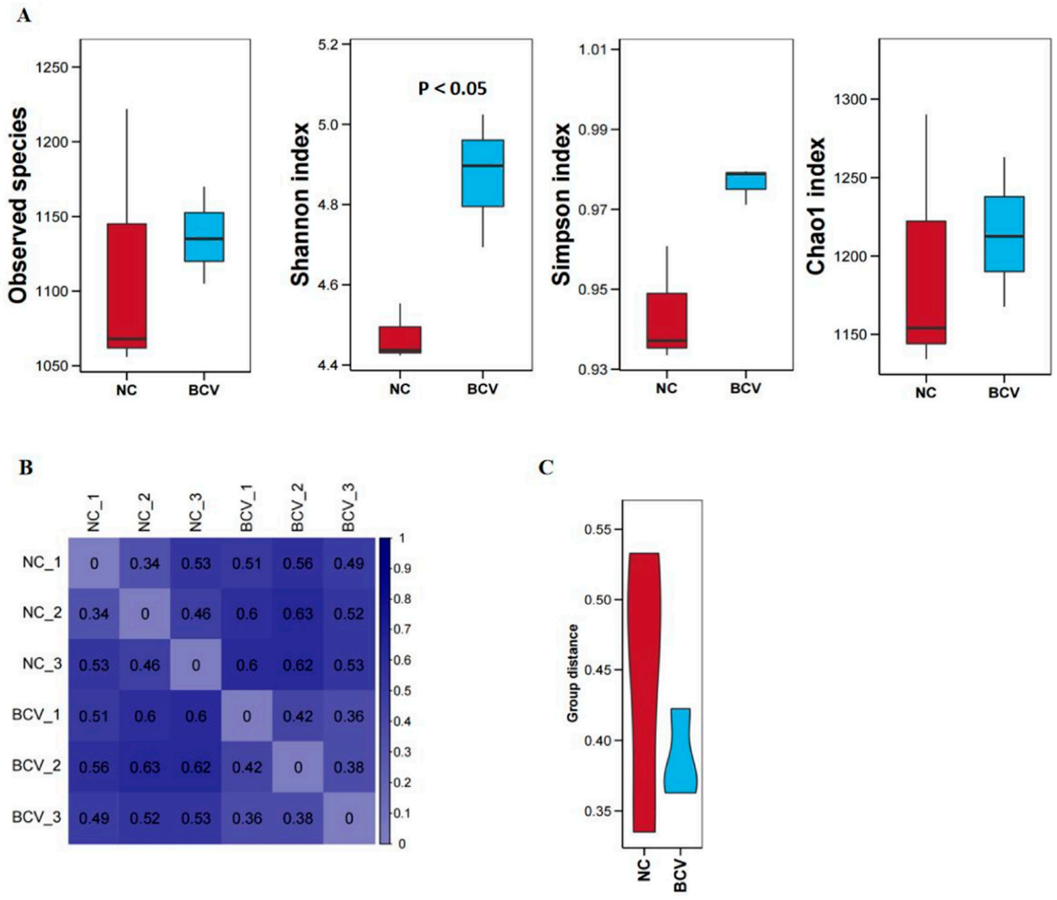


**Supplementary Figure S2. Profiling of the gut microbiota in NC and BCV mice.** **(A)** Box plot displays Observed species, Shannon index, Simpson index and Chao 1 index per group at the operational taxonomic unit (OTU) level. **(B)** Clustered heatmap exhibits the Bray-Curtis dissimilarity at OTU level for each sample, horizontally characterized by different groups. **(C)** Violin plot of group distance between different groups.

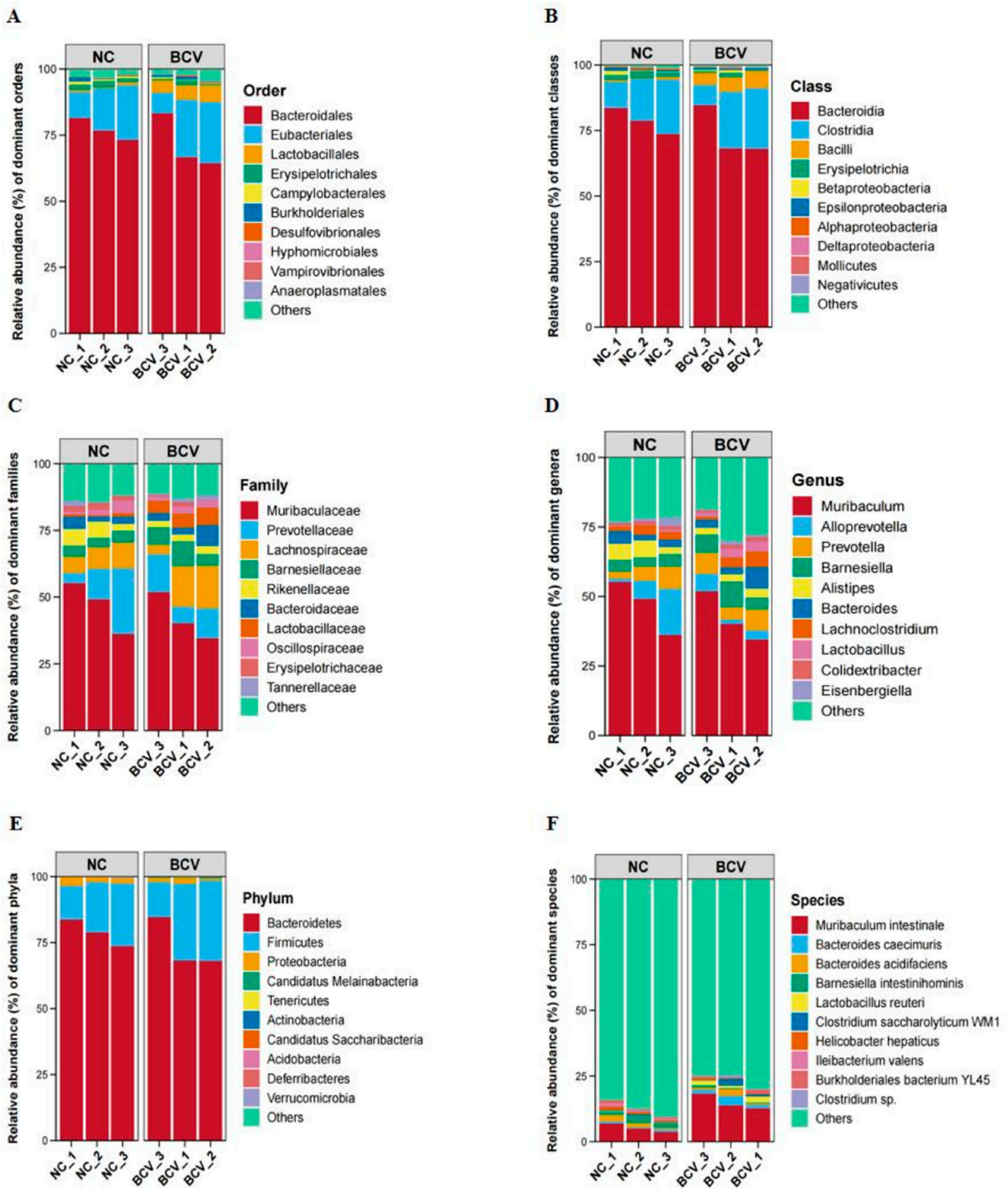


**Supplementary Figure S3. The taxonomic composition of the gut microbiota in NC and BCV mice model. (A-F)** Bar plot displays the taxonomic composition of both groups at the order, class level, family level,genus level, phylum level and species level.

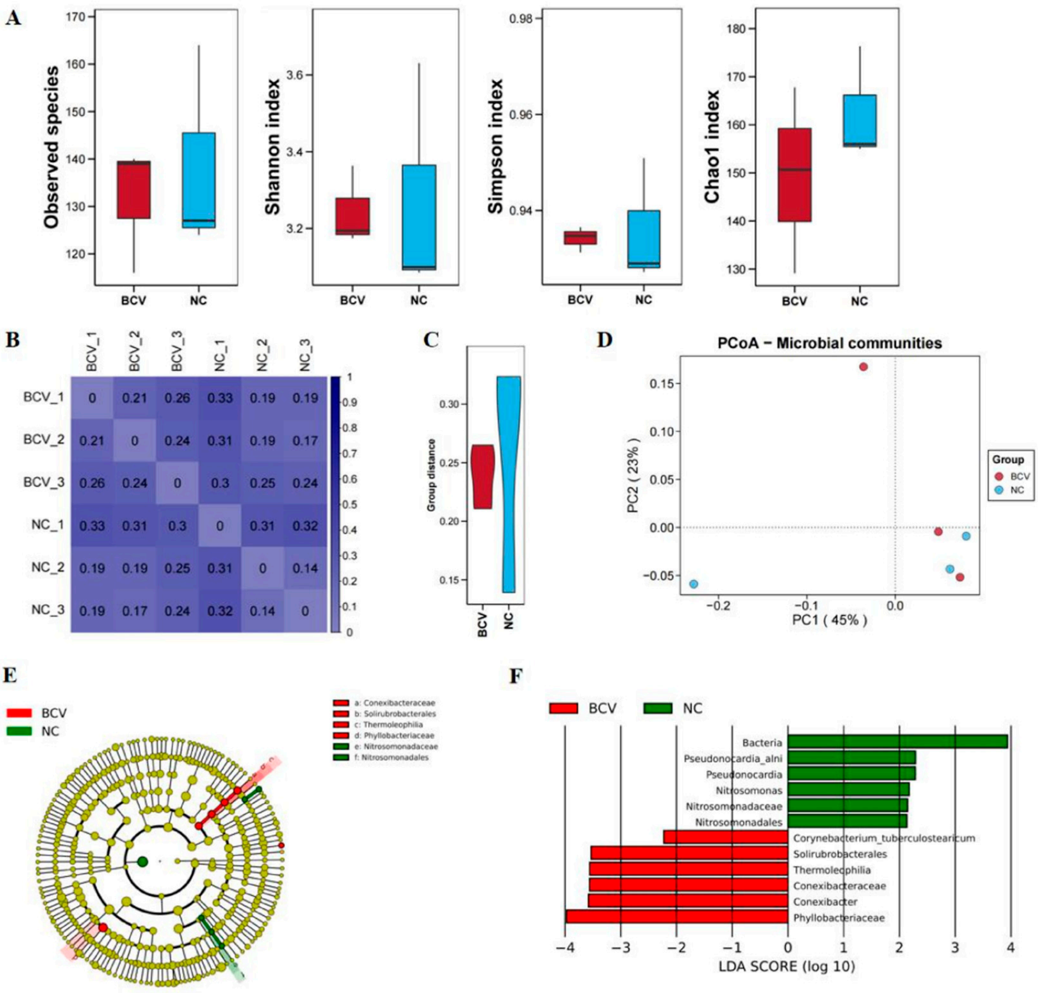


**Supplementary Figure S4. There is little difference in the intratumoral bacteria between NC and BCV mice. (A)** Box plot displays Observed species, Shannon index, Simpson index and Chao 1 index per group at the OTU level. **(B)** Clustered heatmap exhibits the Bray-Curtis dissimilarity. **(C)** Violin plot of group distance between different groups. **(D)** PCoA score plot shows the abundance of species from tumors of NC (blue points) and BCV (red points) mice. **(E)** Taxonomic cladogram from LEfSe depicts taxonomic associations between microbial communities from tumors of NC and BCV mice. **(F)** LDA scores calculated from dissimilarly abundant features between NC and BCV. LDA score>2 is the standard for feature selection.

**Supplementary Table S1. Key resources table.**

| REAGENT or RESOURCE | SOURCE | IDENTIFIER |
|---|---|---|
| APC anti-mouse CD45 | Biolegend | Cat#103112 |
| PerCP/Cyanine5.5 anti-mouse CD3 | Biolegend | Cat#100217 |
| PE/Dazzle™ 594 anti-mouse CD4 | Biolegend | Cat# 100455 |
| Alexa Fluore 700 anti-mouse CD8a | Biolegend | Cat# 100729 |
| Brilliant Violet 650™ anti-mouse CD19 | Biolegend | Cat# 115541 |
| FITC anti-mouse TNF-a | Biolegend | Cat# 506303 |
| Brilliant Violet 605™ anti-mouse IFN-γ | Biolegend | Cat# 505840 |
| PE anti-human/mouse Granzyme B | Biolegend | Cat# 372207 |
| Brilliant Violet 421™ anti-mouse Perforin | Biolegend | Cat# 154319 |
| anti-mouse CD4 | Abcam | Cat# ab237722 |
| anti-mouse CD8 | Bioss | Cat# bs0648r |
| anti-mouse CD19 | Diagbio | Cat# db12393 |
| Alexa Fluor® 700 anti-human CD45 | Biolegend | Cat# 368514 |
| Brilliant Violet 605™ anti-human CD3 | Biolegend | Cat# 317321 |
| APC anti-human CD8 | Biolegend | Cat# 344721 |
| PerCP/Cyanine5.5 anti-human CD19 | Biolegend | Cat# 302229 |
| Brilliant Violet 421™ anti-human TNF-α | Biolegend | Cat# 502931 |
| PE/Dazzle™ 594 anti-human IFN-γ | Biolegend | Cat# 502545 |
| APC/Fire™ 750 anti-human Perforin | Biolegend | Cat# 353317 |
| Collagenase, Type IV | Gibco | Cat# 17104019 |
| Dnase I | Solarbio | Cat# D8071-25mg |
| $C_{26}H_{44}NNaO_7S \cdot xH_2O$ | Sigma-Aldrich | Cat# T9034 |
| Anti-Mouse PD-1 Antibody (RMP1-14) | MCE | Cat# HY-P99144-10mg |
